# Advanced cartilage damage and radiographic osteoarthritis are associated with inferior outcomes and higher rates of conversion to total hip arthroplasty after hip labral reconstructions: A systematic review

**DOI:** 10.1002/jeo2.70812

**Published:** 2026-07-27

**Authors:** Ozgur Basal, Furkan Karakas, James G. Jefferies, Jure Serdar, Baris Kocaoglu, Mahmut Nedim Doral

**Affiliations:** ^1^ Department of Orthopedics and Traumatology Medical Park Gebze Hospital Kocaeli Turkey; ^2^ Faculty of Medicine Dokuz Eylül University İzmir Turkey; ^3^ Department of Orthopedics and Traumatology Maidstone & Tunbridge Wells NHS Trust Kent UK; ^4^ Department of Orthopedics and Traumatology University Hospital Centre Zagreb Zagreb Croatia; ^5^ Department of Orthopedics and Traumatology Acibadem University Faculty of Medicine Istanbul Turkey; ^6^ Department of Orthopedics and Traumatology Medipark Health Center Ankara Turkey

**Keywords:** articular cartilage, femoroacetabular impingement, hip labral reconstruction, outcome predictors, prognostic factors

## Abstract

**Purpose:**

Hip labral reconstruction survival refers to the durability of the reconstructed labrum, typically measured by the rate of freedom from revision surgery or conversion to total hip arthroplasty (THA) over time. This systematic review aimed to identify and combine prognostic factors, particularly those related to cartilage status, graft characteristics, and osteoarthritis, that are associated with functional outcomes and long‐term survivorship after hip labral reconstruction.

**Methods:**

Clinical studies reporting prognostic/exposure factors (e.g., cartilage status, osteoarthritis severity and graft/technique variables) and postoperative outcomes in adult patients undergoing hip labrum reconstruction were searched in PubMed and Scopus databases up to December, 2025. Two reviewers performed the search, data extraction, and risk of bias assessment using ROBINS‐I. Random‐effects meta‐analyses and meta‐regressions were performed when ≥2 studies provided comparable data.

**Results:**

Thirty‐two studies (2252 hips) were included. Patient‐reported outcomes showed significant improvement across cohorts after reconstruction, but survival not requiring arthroplasty varied depending on joint status and technique. In a random‐effects meta‐analysis, the pooled THA conversion rate was 8.8% (95% CI: 6.7%–11.5%). In meta‐regression, a higher prevalence of high‐grade acetabular cartilage damage was associated with increased THA conversion (univariate *p* = 0.035; *β* = 0.0269, *p* = 0.0165 after adjusting for follow‐up time), and longer follow‐up time independently increased the risk of conversion (*β* = 0.0300/month, *p* = 0.0082). In exploratory subgroup analyses, circumferential reconstruction was associated with greater PROM recovery and a lower pooled THA conversion rate than segmental techniques, and allografts were associated with a lower pooled THA conversion rate than autografts; these comparisons are confounded by surgeon experience, institutional volume, and indication, and should be regarded as hypothesis‐generating only.

**Conclusion:**

Outcomes after hip labral reconstruction appear to be associated with preoperative joint status, particularly cartilage damage and radiographic osteoarthritis. These findings are derived from study‐level analyses and should not be interpreted as evidence of independent or causal effects. Apparent differences between graft types and reconstruction configurations are exploratory and likely reflect underlying confounding rather than true treatment effects. Capsular management and concomitant cartilage procedures were not consistently associated with differences in clinical outcomes or THA conversion.

**Level of evidence:**

Level IV, systematic review and meta‐analysis of Level IV–III studies.

**Systematic review registration:** This systematic review was prospectively registered with the International Prospective Register of Systematic Reviews (PROSPERO). Registration number: CRD420251273992. Available at: https://www.crd.york.ac.uk/PROSPERO/view/CRD420251273992

**Abbreviations**
ALAD: acetabular labrum articular disruption
BMI: body mass index
CI: confidence interval
FAI: femoroacetabular impingement
ICC: intraclass correlation coefficient
ICRS: International Cartilage Repair Society
iHOT‐12: International Hip Outcome Tool‐12
MCID: minimal clinically important difference
mHHS: Modified Harris Hip Score
OA: osteoarthritis
OR: odds ratio
PICO: population, intervention, comparison, outcome
PRISMA: Preferred Reporting Items for Systematic Reviews and Meta‐Analyses
PROMs: patient‐reported outcome measures
REML: restricted maximum likelihood
ROBINS‐I: Risk of Bias in Non‐Randomised Studies of Interventions
SD: standard deviation
THA: total hip arthroplasty

## INTRODUCTION

Hip labral reconstruction has become an established joint‐preserving procedure for patients with irreparable labral pathology, most commonly in the context of femoroacetabular impingement (FAI) [7]. Although multiple studies report significant postoperative improvements in patient‐reported outcome measures (PROMs), clinical results remain heterogeneous, and a subset of patients experience persistent symptoms, progression of osteoarthritis (OA), or conversion to total hip arthroplasty (THA) [3, 8, 33]. Identifying factors that determine successful outcomes after labral reconstruction is therefore of critical importance.

The success of hip‐preserving surgical procedures is highly dependent on patient selection and the underlying anatomy of the hip. Borderline acetabular dysplasia continues to be a problematic condition with suboptimal patient‐reported results and continuous discussion over the ideal joint‐preserving approach, including periacetabular osteotomy versus hip arthroscopy [17, 34]. In contrast, the arthroscopic treatment of well‐defined FAI has shown persistent functional improvement at a minimum of 5‐year follow‐up [38]. These results altogether highlight the relevance of preoperative joint condition and proper patient selection.

The presence of an irreparable or inadequate acetabular labrum is the primary aetiological factor associated with hip labral reconstruction. Hip labral tears as a result of chronic degeneration concomitant with FAI, complex or mixed‐type tears (Seldes types 1 and 2), or previous failed labral repairs are the most common etiologies [14]. Pathological ossification or calcification of the labrum compromises its functionality and impedes the regeneration of native tissue, thereby necessitating surgical reconstruction [2]. A hypoplastic labrum is characterised by inadequate tissue thickness, frequently measuring less than two to three millimetres, and a compromised suction seal. Such a labrum is deemed nonfunctional and may warrant reconstruction, especially if it fails to withstand axial distraction under appropriate force [25].

Long‐term rates of conversion to THA after hip labral reconstruction vary widely depending on patient selection, surgical technique, and follow‐up duration. Systematic reviews and large cohort studies report conversion rates ranging from approximately 1.6% to 27% at mid‐ to long‐term follow‐up, with most studies clustering between 5% and 15% at 2–10 years [8, 33]. For instance, a systematic review with minimum 5‐year follow‐up found conversion rates of 1.6%–27% after labral reconstruction, while a 10‐year follow‐up study reported a 27% conversion rate overall, but only 10% in patients with >2 mm joint space [29].

Graft‐related and technical factors, including graft type and fixation characteristics, represent potentially modifiable variables that may influence restoration of labral function and joint biomechanics [27]. There remain important gaps regarding the optimal graft selection, chondral status, and standardisation of reconstruction techniques. Despite increasing surgical adoption, the prognostic value of these factors has not been systematically analysed [35].

The primary aim of this study was to systematically evaluate the prognostic impact of cartilage pathology, radiographic OA severity, and graft‐related factors on PROMs, OA progression, and conversion to THA following hip labral reconstruction in adults. Secondary aims were to analyse graft‐specific functional outcomes and survivorship, compare autograft and allograft reconstruction with respect to THA conversion risk, and identify evidence gaps related to graft selection and technical variables that may inform patient selection and joint‐preserving strategies. The hypothesis of this systematic review is that cartilage damage and radiographic OA are associated with inferior outcomes and higher rates of conversion to THA after hip labral reconstructions.

## METHODS

### Protocol and registration

Following the PRISMA (Preferred Reporting Items for Systematic Reviews and Meta‐Analyses)‐guidelines, a systematic review was designed to evaluate intraarticular and surgical prognostic factors affecting outcomes after hip labrum reconstruction in adults. The systematic review was registered in PROSPERO and conducted in accordance with the PRISMA 2020 guidelines. (Registration No.: CRD420251273992).

### Eligibility criteria

The eligibility criteria were defined according to the Population, Intervention (Exposure), Comparison, Outcome, and Study design (PICO) framework.

#### Population

Studies of adult patients (≥18 years old) undergoing arthroscopic hip labral reconstruction for labral tears, typically in the context of FAI were included. Patients treated with isolated labral repair or debridement (without reconstruction) were excluded, as were paediatric populations and patients with developmental hip dysplasia not managed with reconstruction.

#### Interventions/exposures

The review focused on prognostic factors present at the time of labral reconstruction, specifically: (1) concomitant articular cartilage damage of the hip (acetabular and/or femoral head lesions, graded by Outerbridge/ALAD/ICRS classifications); (2) pre‐existing radiographic OA (e.g., Tönnis grade >0 or evidence of joint space narrowing); and (3) graft‐related and technical variables, including graft type (such as iliotibial band allograft vs. hamstring autograft), graft fixation method, and graft size.

#### Comparators

No separate control group was required for inclusion. Instead, subgroup analyses were performed within the labral reconstruction cohort. Outcomes were compared between subgroups of patients stratified by the prognostic factors of interest (e.g., those with vs. without chondral damage, low‐grade vs. high‐grade chondral lesions, presence vs. absence of concomitant cartilage repair procedures, different preoperative Tönnis grades, and different graft types).

#### Study designs

Both randomised controlled trials and observational studies in English language were included in the systematic review (prospective or retrospective cohort studies and case series with ≥ 10 patients). Small case reports with fewer than 10 patients, biomechanical or cadaveric studies, expert opinion pieces, editorials, and prior systematic reviews were excluded. Studies were eligible for inclusion, as long as they reported postoperative outcomes following hip labral reconstruction. Included studies were required to report at least one of the relevant outcome measures described below.

### Literature search strategy

A comprehensive literature search was performed to identify all relevant studies on hip labral reconstruction outcomes related to cartilage, OA, or graft factors. From the outset, we searched PubMed (MEDLINE) and Scopus using database‐adapted combinations of hip/labrum/reconstruction terms (TITLE‐ABS‐KEY(hip OR ‘hip joint’ OR acetabular) AND TITLE‐ABS‐KEY (labrum OR labral OR ‘acetabular labrum’) AND TITLE‐ABS‐KEY(reconstruct* OR ‘labral reconstruction’ OR graft* OR augmentation)) and supported this with prospective citation searches of the included studies. The search was limited to studies published in the English language, and only full‐text published articles were considered (conference abstracts, dissertations, and other unpublished data were not included). We also performed forward citation tracking (snowballing) by examining articles that cited the included studies to identify any additional publications meeting the inclusion criteria. All database search results were imported into reference management software, and duplicate records were removed.

**Figure 1:**
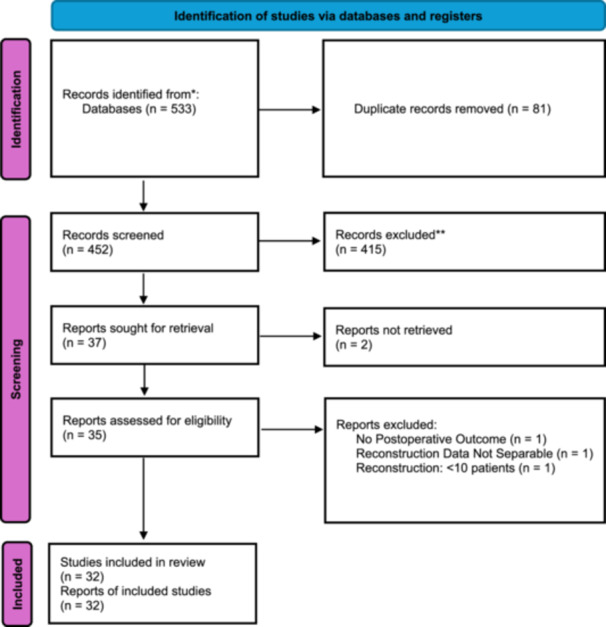
PRISMA 2020 flow diagram of study selection for review process.

A title/abstract search commenced on 21 December 2025; study selection resulted in 533 records being identified, 81 duplicate records were removed. Titles and abstracts of 452 records were screened, and 415 were excluded at the title/abstract stage. Full‐text reports were sought for 37 articles; two reports could not be retrieved. Of the 35 reports assessed for eligibility, three were excluded due to lack of postoperative outcomes (*n* = 1), non‐separable reconstruction data (*n* = 1), or insufficient sample size (<10 patients; *n* = 1). Ultimately, 32 studies were included (Figure 1).

### Study selection

Study selection was carried out in two stages. First, two reviewers independently screened all retrieved titles and abstracts for potential eligibility. Studies that clearly did not meet inclusion criteria (e.g., wrong population or intervention, non‐clinical studies) were excluded at this stage. Next, the full texts of all remaining articles were obtained and assessed independently by the same two reviewers against the eligibility criteria. Any disagreements or discrepancies regarding study inclusion were resolved through discussion and consensus; a third reviewer was available to adjudicate if necessary. The study selection process, including the number of articles screened, duplicates removed, and reasons for exclusion at the full‐text stage, are summarised in a PRISMA flow diagram.

### Data extraction

Two reviewers independently screened and extracted data using a standardised form; disagreements were resolved through consensus; study authors were not contacted. Extracted data included study characteristics (author, year, study design, sample size and hip size), patient demographics (age, sex and BMI), details of the surgical procedure (labral reconstruction technique, graft type and source, concomitant procedures), the presence and grade of any chondral lesions or OA at baseline, follow‐up duration, and all relevant outcome measures. For continuous outcomes reported only graphically, approximate values were extracted from graphs when possible. Any differences between reviewers in the extracted data were resolved by consensus. Only published data were used; original study authors were not contacted for additional information or missing data. Data synthesis was primarily performed narratively using structured tables; when sufficiently homogeneous data were available, a randomised effects meta‐analysis was performed by combining continuous results as mean differences/standardised mean differences and binary results as risk ratios/odds ratios (ORs) (95% confidence interval [CI]), assessing heterogeneity with *I* ^2^ and Cochran's Q, and (where possible) performing subgroup analyses according to cartilage severity, OA grade, and graft/technique factors.

Flow diagram illustrating the identification, screening, eligibility assessment, and inclusion of studies according to the PRISMA 2020 guidelines. A total of 533 records were identified through database searching. After removal of 81 duplicate records, 452 records were screened at the title and abstract level, of which 415 were excluded. Full‐text reports were sought for 37 articles; two reports could not be retrieved, and three studies were excluded due to lack of postoperative outcomes (*n* = 1), non‐separable reconstruction data (*n* = 1), or insufficient sample size (<10 patients; *n* = 1). Ultimately, 32 studies were included in the qualitative synthesis, of which 32 were included in the quantitative synthesis (meta‐analysis).

### Risk of bias assessment

**Figure 2:**
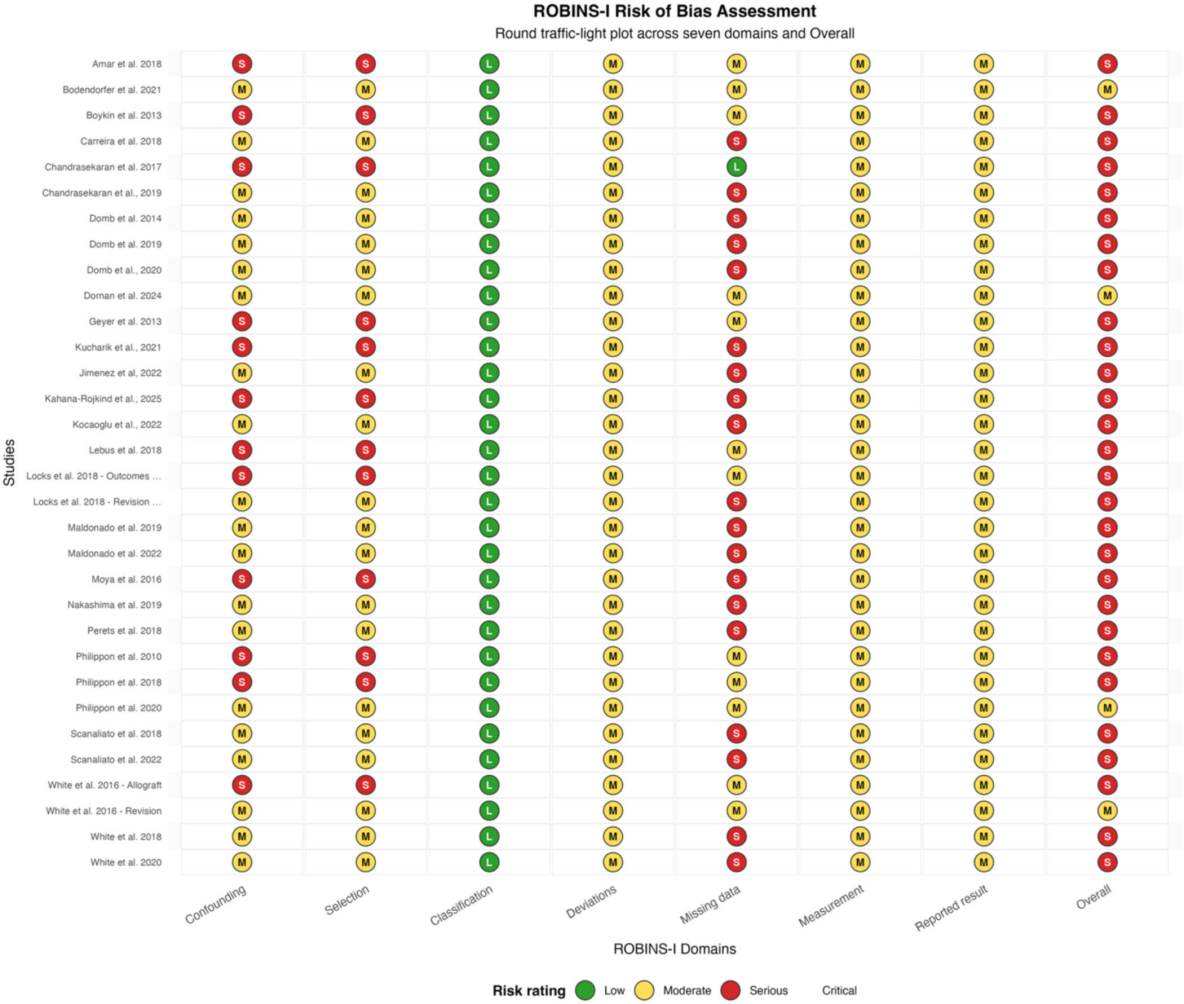
ROBINS‐I risk of bias assessment across included studies traffic‐light plot illustrating risk of bias across seven domains (green: low risk; yellow: moderate risk; red: serious risk). Most studies showed moderate to serious risk in confounding, selection, and selective reporting domains. ROBINS‐I, Risk of Bias in Non‐Randomised Trials‐Interventions.

Risk of bias was assessed using validated tools specific to each study design. Following a formal literature search, no randomised controlled trials meeting the inclusion criteria were found. All 32 observational studies were assessed using the ROBINS‐I (Risk of Bias in Non‐Randomised Trials‐Interventions) tool, which examines bias in seven domains: (1) confounding factors, (2) selection of participants, (3) classification of interventions, (4) deviations from intended interventions, (5) missing data, (6) measurement of outcomes, and (7) selection of reported outcome. Each domain was assessed as having a low, moderate, serious, or critical risk of bias. An overall risk of bias assessment for each study was derived from the domain‐level assessments. Assessments were performed independently by two reviewers, and differences were resolved by consensus with a third reviewer. Inter‐reviewer reliability was excellent (intraclass correlation coefficient [ICC(2,1)] = 0.90). Figure 2 illustrates the risk of bias across the included studies [1].

A small proportion of the studies were determined to carry a moderate risk of bias, primarily due to residual confounding factors and the lack of blinding in outcome assessment specific to observational designs. A higher number of studies demonstrated a significant risk of bias, which is frequently linked to incomplete outcome data or participant selection. There were no studies deemed to have a critical risk of bias. These results highlight the need for careful interpretation of combined estimates, particularly for outcomes that are especially sensitive to confounding factors such as long‐term survival and OA progression.

### Data synthesis and statistical analysis

Data synthesis was performed at the study level. Continuous outcomes (e.g., changes in PROMs) were summarised using weighted means when variance data were unavailable and pooled using mean differences or standardised mean differences with 95% CIs, as appropriate. Binary outcomes, including conversion to THA, were pooled using generalised linear mixed‐effects random‐effects models on the log‐odds scale.

Random‐effects models were fitted using restricted maximum likelihood (REML) estimation of between‐study variance (τ^2^), which is preferred over alternative estimators (e.g., DerSimonian–Laird) due to reduced bias under conditions of heterogeneity [42]. The Hartung–Knapp–Sidik–Jonkman adjustment was applied to all pooled estimates to provide more conservative CIs, particularly when the number of studies was limited.

Between‐study heterogeneity was assessed using the *I* ^2^ statistic, τ^2^, and Cochran's Q test. Predefined subgroup analyses were conducted according to graft source (autograft vs. allograft), graft configuration (segmental vs. circumferential), cartilage procedure type (microfracture vs. chondroplasty), and cartilage severity, where data were available. Study‐level meta‐regression analyses were performed to explore associations between THA conversion and potential moderators, including mean follow‐up time, the proportion of high‐grade cartilage lesions, and the proportion of concurrent cartilage procedures; these analyses were interpreted cautiously given their ecological nature.

Sensitivity analyses included leave‐one‐out analyses and cumulative meta‐analysis. Small‐study effects were assessed using funnel plots and Egger's test when ≥10 studies were available. All analyses were performed in R (RStudio, Vienna, Austria) using the *meta* and *metafor* packages, with statistical significance set at *p* < 0.05.

### Handling of missing data

Missing data were addressed according to established procedures for systematic reviews and meta‐analyses that include study‐level data. For continuous outcomes, if means were reported without accompanying measures of variance (such as standard deviations [SDs], standard errors, or CIs), SDs were estimated using validated estimation techniques. These methods involved calculating SDs derived from reported ranges, interquartile ranges, or *p*‐values when such data were available. Conversely, if these statistics were unavailable, the median SD from other studies with comparable populations and outcomes was used for estimation, and sensitivity analyses were performed to assess the impact of estimates on combined estimates. For binary outcomes, studies reporting zero events in a group received a continuum correction of 0.5 to allow for log‐likelihood transformation. When essential prognostic data (e.g., cartilage grade distribution and graft details) were not reported, the study was excluded from the relevant subgroup or meta‐regression analyses. We did not contact study authors for additional data; all analyses are based solely on published information. The potential impact of missing data on the study results was acknowledged as a limitation in Discussion section.

### Unit of analysis

The unit of analysis was the aggregate outcome at the study level. For studies reporting multiple treatment arms (e.g., autograft and allograft cohorts within the same study), each arm was treated as an independent comparison in subgroup analyses when patient cohorts were clearly non‐overlapping.

When outcomes were reported at multiple time points, the longest available follow‐up was selected for the primary analysis to capture clinically relevant endpoints such as conversion to THA. For studies including bilateral procedures, outcomes were analysed per hip when explicitly reported; otherwise, patient‐level data were used.

To minimise potential duplication, studies originating from the same institution with overlapping enrolment periods were carefully evaluated based on author group, study period, and cohort characteristics. In cases of suspected overlap, the most comprehensive data set—defined as the study with the largest sample size, longest follow‐up, or most complete outcome reporting—was retained.

Given the concentration of studies from high‐volume centres, residual overlap could not be completely excluded. To assess the robustness of the findings, sensitivity analyses were performed by sequentially excluding influential studies and by examining the consistency of results across study subgroups. These analyses demonstrated that no single study disproportionately affected the pooled estimates.

## RESULTS

### Study characteristics

Across the 32 included studies, 26 were retrospective, four were prospective, and two did not specify a prospective/retrospective design. In total, 2235 patients and 2252 hips were reported (hip counts exceeded patient counts because several studies reported more hips than patients). Because reporting was inconsistent, the number of hips contributing to each analysis varied by outcome availability. Using hip‐weighted pooled summaries, the mean age was 37.0 years (study mean range, 27.0–52.6; reported min–max across studies providing ranges, 14.9–76.0). The hip‐weighted proportion of male participants was 44.3% (range across studies, 19.6%–100%). BMI was reported in 21/32 studies, representing 1474/2252 hips (65.5%), with a hip‐weighted mean BMI of 25.1 kg/m^2^ (study mean range, 23.27–27.8; reported min–max range, 17.0–38.7). Follow‐up duration showed a hip‐weighted mean of 46.2 months (study mean range, 18.0–132.0; reported min–max range, 12–174). Baseline patient characteristics are summarised in Table 1.

**Table 1:** Baseline characteristics included studies evaluating hip labral reconstruction and related predictors.

Study_ID	Study design	Sample size (*N*)	Hip size (*N*)	Age, years (mean ± SD (range))	% Male	BMI, kg/m^2^ (mean ± SD (range))	Follow‐up month (mean ± SD (range))
Amar et al. [1]	Retrospective case series	31	31	42 (22–68)	59.1% (13/22)	NR	36.2 (24–72)
Bodendorfer et al. [3]	Retrospective cohort	55	55	34.4 ± 9.7	32.1%	24.1 ± 3.8	24.8
Boykin et al. [4]	Retrospective case series	23	23	28 (19–41)	100%	NR	41.4 (20–74)
Carreira et al. [5]	Prospective cohort	31	31	43.7 ± 9.2 (20–66)	35.5%	24.2 ± 4.0 (17–31)	31.6 ± 7.1 (24–46)
Chandrasekaran et al. [6]	Retrospective case series	22	22	32.2 ± 9.8 (15–45)	36.4%	25.2 ± 3.94	29.1 ± 6.9
Chandrasekaran et al. [7]	Retrospective comparative	34	34	37.3 ± 12.2 (15.5–61.9)	52.9%	26.9 ± 4.7 (19.3–38.2)	36.8 ± 13.8 (24.0–72.0)
Domb et al. [10]	Retrospective cohort	11	11	33.0 ± 9.9 (18.0–44.9)	63.6%	24.5 ± 3.0 (21.5–30.5)	26.4 ± 3.6 (24.0–32.4)
Domb et al. [9]	Retrospective cohort	23	23	35.2 ± 11.9 (15.5–61.9)	47.8%	24.8 ± 4.0 (18.1–32.5)	67.2 ± 7.7 (60.0–89.3)
Domb et al. [11]	Retrospective case–control	37	37	45.6 ± 11.6	48.6%	27.2 ± 4.6	25.5 ± 1.6
Dornan et al. [12]	Prospective comparative study	150	150	38 ± 12 (18–71)	47.3%	24.7 ± 3.6 (17.8–37.4)	76.8 ± 40.8 (25.2–174)
Geyer et al. [13]	Retrospective case series	75	76	38.5 (18–64)	56%	25 (19–36)	49 (36–70)
Kucharik et al. [19]	Retrospective case series	94	97	39.0 (36.8–41.2)	49.5%	25.8 (24.9–26.7)	28.2 (26.0–30.4)
Jimenez et al. [15]	Retrospective cohort	46	47	29.6 ± 9.7	33%	25.4 ± 4.5	31.7 ± 9.3
Kahana‐Rojkind et al. [16]	Retrospective comparative case Series	150	150	33.1 ± 10.7	41.3%	25.4 ± 4.7	52.5 ± 29.3
Kocaoglu et al. [18]	Prospective cohort	42	42	32.6 ± 7.0 (21–54)	33.3%	NR	33.6 ± 5
Lebus et al. [20]	Retrospective case series	368	368	33.8 (16–69)	55.1%	25.0 (22.5–28.2)	44.4 (24.0–135.6)
Locks et al. [22] ‐ Outcomes …	Retrospective case series	11	11	35.4 ± 11.1 (20–51)	54.5%	NR	65.0 ± 44.3 (12–134)
Locks et al. [21] ‐ Revision …	Retrospective comparative	36	36	33 ± 14	22.2%	NR	43.2 ± 12.0
Maldonado et al. [24]	Retrospective comparative cohort	29	29	36.3 ± 11.6 (17.9–56.1)	55.2%	27.7 ± 5.1 (17.3–38.7)	38.5 ± 15.3 (24.0–72.0)
Maldonado et al. [23]	Retrospective cohort	41	41	37.5 ± 10.4 (34.4–40.7)	61%	27.8 ± 5.8 (26.1–29.6)	64.4 ± 24.1 (57–71.8)
Moya et al. [26]	Case series	20	20	34.5 (28–46)	80%	NR	61.2 (24–90)
Nakashima et al. [28]	Case–control study	25	25	52.6 ± 15.0 (20–76)	72%	24.3 ± 2.5 (19.6–29.4)	37.0 ± 13
Perets et al. [29]	Retrospective cohort	15	15	27.0 ± 8.2 (17.8–40.7)	33.3%	23.8 ± 3.2 (18.1–29.9)	36.6 ± 16.9 (21.6–68.2)
Philippon et al. [32]	Retrospective case series	47	47	37 (18–55)	68%	24 (19–31)	18 (12–32)
Philippon et al. [31]	Retrospective comparative case series	66	66	29 ± 10	36.4%	NR	41 ± 16
Philippon et al. [30]	Retrospective case series	89	91	38.7 ± 11.4 (18–65)	62.2%	24.3 ± 3.9	132 (120–156)
Scanaliato et al. [36]	Retrospective cohort	63	63	43.4 ± 10.7	41.3%	24.6 ± 3.8	24 ± 1.9 (22–26)
Scanaliato et al. [37]	Retrospective cohort	62	62	38.3 ± 11.2 (22–70)	37.10%	23.27 ± 3.3	60.4 ± 1.5
White et al. [42] ‐ Allograft	Prospective case series	142	152	39 (16–58)	45.0%	NR	28 (24–39)
White et al. [39] ‐ Revision	Retrospective comparative cohort	98	98	34.6 ± 10.2	27%	NR	28.8 (24‐48)
White et al. [40]	Retrospective comparative cohort	29	29	33.3 ± 11.0 (14.9–51.6)	20.7%	NR	40 (22–61)
White et al. [41]	Retrospective comparative	270	270	42.5 ± 8.1 (30–65)	19.6%	NR	44.6 ± 17.8
Overall		2235	2252	37.0	44.3%	25.1	46.2

Abbreviations: BMI, body mass index; NR, not reported; SD, standard deviation.

Regarding institutional and geographic distribution, a substantial proportion of evidence originated from a small number of high‐volume US centres (American Hip Institute: 8/32 [25.0%]; Steadman Philippon Research Group: 5/32 [15.6%]; Colorado Center for Orthopaedic Excellence: 4/32 [12.5%]), with the remaining 15/32 (46.9%) from independent institutions. Overall, 28/32 studies (87.5%) were conducted in the United States, contributing 2100 patients and 2117 hips, whereas 4/32 (12.5%) came from non‐US centres (Turkiye, Israel, Japan and Spain), contributing 118 patients and 118 hips. Detailed surgical characteristics and outcomes are summarised in Table 2. The dominance of a limited number of high‐volume centres raises the possibility that reported outcomes may reflect center‐specific expertise and surgical philosophy, potentially limiting generalisability.

**Table 2:** Surgical characteristics, concomitant cartilage procedures and survivorship outcomes of included studies on hip labral reconstruction.

Study (author, year)	Sample size (hips)	Primary/revision	Configuration	Graft (auto/allo; source)	Fixation	Capsular management	Concomitant cartilage procedure	OA progression (n/N)	THA conversion (n/N)
Amar et al. [1]	31	Primary 45.2% (14/31); Revision 54.8% (17/31)	Segmental	Autograft (indirect head rectus femoris)	Anchors (Knotted)	T‐capsulotomy with or without capsular closure	Debridement	NR	2/31
Bodendorfer et al. [3]	55	Revision 100%	Segmental (26/55), Circumferential (29/55)	NR (Multicenter)	NR	Capsular repair (T‐capsulotomy repaired)	Chondroplasty (68%), Microfracture (4%)	NR	0/55
Boykin et al. [4]	23	Primary 52.17% (12/23); Revision 47.8% (11/23)	Segmental	Autograft (iliotibial band)	Anchors (Knotted)	NR	Microfracture	NR	2/23 (8.7%)
Carreira et al. [5]	31	Revision 19.4%	Segmental	Allograft (fascia lata)	Anchors (Knotted)	Closure 67.8%, Plication 29.0%, Release 3.2%	Acetabular Chondroplasty (48.4%), Acetabular Microfracture (29.0%)	NR	4/31 (12.9%)
Chandrasekaran et al. [6]	22	Primary 45% (10/22); Revision 55% (12/22)	Segmental	Mixed (semitendinosus allograft or gracilis autograft)	Knotless anchors (PushLock)	Repair (32%); Release (68%)	Acetabular Chondroplasty (40.9%), Acetabular Microfracture (13.6%), Femoral Head Chondroplasty (13.6%)	NR	1/22
Chandrasekaran et al. [7]	34	Primary 100%	Segmental	Autograft (18/34) 52.9% (gracilis autograft); Allograft (16/34) 47.1%) (semitendinosus allograft)	Anchors (PushLock; knotless)	Repair/plication 14; release 20	Acetabular microfracture (5.9%)	NR	4/34
Domb et al. [10]	11	Primary 45.5% (5/11); Revision 54.5% (6/11)	Segmental	Autograft (Gracilis tendon)	Knotless anchors (PushLock)	Repair/Closure	NR	NR	0/11
Domb et al. [9]	23	Primary 73.9% (17/23); Revision 26.1% (6/23)	Segmental	NR	Knotless anchors (PushLock)	Capsular repair 10; Release 13	Acetabular microfracture (8.7%)	NR	3/23 (13.0%)
Domb et al. [11]	37	Primary 100%	Circumferential	Allograft ‐ Anterior tibialis tendon	Knotless anchors	Repair 16/37; Capsulotomy without repair 21/37	Acetabular microfracture 6; femoral head microfracture 1	NR	2/37
Dornan et al. [12]	150	Primary 100%	NR	Not specified	NR	NR	Acetabular microfracture 29 (22%), femoral head microfracture 7 (5.4%)	NR	26/129
Geyer et al. [13]	76	Primary 51% (39/76); Revision 49% (37/76)	Segmental	Autograft (Iliotibial band)	Anchors (suture)	NR	Microfracture 22 (29%)	NR	19/76 (25%)
Kucharik et al.[19]	97	Primary 100%	Segmental/augmentation‐type	Autograft capsule	Anchors	No repair	Microfracture 2 (2.1%)	NR	2/97
Jimenez et al. [15]	47	Revision	Segmental 28/30; Circumferential 2/30	Allograft 29/30; Autograft 1/30	Knotless pull‐through	Capsular repair 31/47 (66%)	Acetabular microfracture 2.1% (1/47)	Tönnis 0‐ > 1 2/47 hips (4%)	1/47 (2.1%)
Kahana‐Rojkind et al. [16]	150	Primary 50% 75/150; Revision 50% 75/150.	NR	Allograft	Anchors (Knotless pull‐through	Repair 97/150; Plication 31/150; Release 22/150	Microfracture 17/150 (11.3%)	NR	14/150 (9.3%)
Kocaoglu et al. [18]	42	Primary 100%	Side‐to‐side anastomosis; anchors at 1‐cm intervals	Autograft (Iliotibial) (20/42); allograft (tibialis anterior (22/42)	Anchors (PushLock)	Capsule closure 39/42	Debridement	0/42	0/42
Lebus et al. [20]	368	Primary 38% (122/317); Revision 62% (195/317)	NR	Autograft (iliotibial band)	Suture anchors	NR	Microfracture	NR	42/368
Locks et al. [22] ‐ Outcomes…	11	NR	Segmental	Autograft ((capsule tissue (8/11); rectus femoris tendon (3/11))	Anchors	NR	Acetabular chondral debridement (8/11)	NR	0/11
Locks et al. [21] ‐ Revision…	36	Revision 100% (28/28)	Segmental	Autograft (Iliotibial band)	Anchors	Capsular closure	Microfracture performed in some	NR	4/28
Maldonado et al. [24]	29	Primary 100%	Segmental	Allograft 17/29 (58.6%) ‐ Semitendinosus; Autograft 12/29 (41.4%) ‐ Semitendinosus	Anchors (Knotless & PushLock)	Plication 14/29; Capsular release 15/29	Acetabular microfracture (13.8%, 4/29)	NR	4/29
Maldonado et al. [23]	41	Primary 100%	Segmental	Autograft (Hamstring) 15/41 (36.6%); Allograft (Hamstring) 26/41 (63.4%)	Anchors (PushLock)	Interportal capsulotomy without repair 25/41; capsular repair 16/41; capsular plication 4/41	Acetabular microfracture (17.1%)	NR	7/41
Moya et al. [26]	20	NR	Segmental	Peroneus lateralis brevis tendon allograft (11/20), Labrum allograft (9/20)	Suture Anchors	Repair/closure	NR	1/20	1/20
Nakashima et al. [28]	25	Primary 100%	Segmental	Autograft ‐ Iliotibial band	Suture Anchors	Repair/closure	Microfracture 4/25 (16%)	NR	3/25
Perets et al. [29]	15	Revision 100%	Segmental	Both autograft and allograft; Gracilis autograft (initially); Semitendinosus allograft (later)	PushLock anteriorly, SutureTak posteriorly; remainder knotless SutureTak anchors	Capsular repair 10/15, Capsular release 5/15	Acetabular microfracture 26.7% (4/15)	NR	1/15
Philippon et al. [32]	47	Primary 51% (24/47); Revision 49% (23/47)	Segmental	Autograft (Iliotibial band)	Suture Anchors	NR	NR	NR	4/47
Philippon et al. [31]	66	Revision 100%	Segmental	Autograft (Iliotibial band)	Suture anchors	Repair/Closure	Acetabular chondroplasty 40/66; Femoral head chondroplasty 27/66; Acetabular microfracture 1/66;	NR	3/66
Philippon et al. [30]	91	Primary 50%; Revision 50%	Segmental	Autograft (liotibial band)	Suture anchors	NR	Microfracture 16 hips; (4both + 2 femoral only + 10 acetabular only)	NR	22/91
Scanaliato et al. [36]	63	Primary 100%	Circumferential/Complete	Allograft (Fascia lata)	Suture anchors	Repair/Plication 100%	Acetabular chondroplasty 56/63 (88.9%); Femoral head chondroplasty 9/63 (14.3%); Unspecified chondroplasty 2/63 (3.2%); Acetabular microfracture 1/63 (1.6%); Drilling of subchondral cyst 1/63 (1.6%)	NR	2/63
Scanaliato et al. [37]	62	Primary 100%	Circumferential/Complete	Allograft ‐ Fascia lata	Anchors	Interportal capsulotomy	Acetabular chondroplasty (90.3% [56/62])	NR	1/62
White et al. [42] ‐ Allograft	152	Primary 75.5% (99/131); Revision 24.5% (32/131)	Segmental	Allograft (Iliotibial band)	Anchors	Repair/Closure	Microfracture 27, Chondroplasty 30	13/131	13/152
White et al. [39] ‐ Revision	98	Revision 100%	Circumferential/Complete	Allograft (Iliotibial band)	Anchors	NR preserved	Microfracture 6% (6/98); Chondroplasty 42% (41/98)	NR	6/98
White et al. [40]	29	Primary 100%	Circumferential/Complete	Allograft (Iliotibial band)	Anchors	NR	NR	NR	0/29
White et al. [41]	270	Primary 100%	Circumferential/Complete	Allograft (Iliotibial band)	Anchors	Capsule preserved	Chondroplasty	NR	10/230
Overall	2252	Primary: 1401/2221 (63.1%) Revision: 820/2221 (36.9%)	Segmental: 935/1567 (59.7%) Circumferential/complete: 590/1567 (37.7%) Other: 42/1567 (2.7%)	Allograft: 1022/2007 (50.9%) Autograft: 948/2007 (47.2%) Mixed: 37/2007 (1.8%)					203/2183 (9.3%)

Abbreviations: NR, not reported; OA, osteoarthritis; THA, total hip arthroplasty.

The demographic characteristics and follow‐up periods of the 32 studies included in this systematic review are summarised in the demographic table. The evidence base was predominantly retrospective (26 of the 32 studies were explicitly labelled as retrospective), with 4 prospective studies and 2 studies reported as case series/case‐control designs without a clear prospective/retrospective classification. In all studies, the total reported sample size was 2235 patients and the total reported number of surgical units was 2252 hips; the number of hips slightly exceeded the number of patients, as five studies reported more hips than patients; this is consistent with bilateral cases and/or differences in reporting. Overall, patients were young to middle‐aged adults: the hip‐weighted mean age was 37.0 years, and study mean ages ranged from 27.0 to 52.6 years; ages ranged from 14.9 to 76.0 years among studies reporting ranges. The gender distribution favoured women; The hip‐weighted mean was 44.3% male (study‐level values ranged from 19.6% to 100%). Body Mass Index (BMI) reporting was inconsistent; BMI was available in 21 of 32 studies representing 1474 (65.5%) of 2252 hips, and the hip‐weighted mean BMI among studies reporting BMI was 25.1 kg/m^2^ (study mean BMI 23.27–27.8 kg/m^2^; reported ranges 17.0–38.7 kg/m^2^). Follow‐up duration was highly variable: the hip‐weighted mean follow‐up duration was 46.2 months, while study mean follow‐up duration ranged from 18.0 to 132.0 months and reported follow‐up durations ranged from 12–174 months.

In the included studies, labrum reconstruction cohorts consisted of 2252 hips (32 cohorts). Overall, primary/revision status was reported for 2221 hips; of these, 1401 (63.1%) were primary reconstructions and 820 (36.9%) were revision reconstructions; primary/revision status was not reported for the remaining hips. Regarding reconstruction configuration, reporting was available for 1567 hips; segmental reconstruction was predominant (59.7%, 935 hips), followed by circumferential/complete reconstruction (37.7%, 590 hips), and other techniques were rare (2.7%, 42 hips); configuration was not reported for the remaining hips. For graft selection, graft type was specified for 2007 hips, with allografts used slightly more frequently than autografts (1022 hips, 50.9% vs. 948 hips, 47.2%); mixed graft use was rare (37 hips, 1.8%), and graft type/source was not specified for the remaining hips. Fixation method was reported for 2047 hips, with anchorage‐based fixation (including knotted and knotless anchorage constructions) being the most common, while traction/looped knotless techniques were described in a smaller number of cohorts. Reporting of radiographic OA progression was limited; among cohorts with extractable data, OA progression occurred in 16 of 240 hips (6.7%). In contrast, THA conversion has been reported more consistently, with 203 conversions occurring among 2183 reported hips, indicating an overall THA conversion rate of 9.3% (203/2,183). When combined using a random effects model, the estimated THA conversion rate was found to be 8.85% (95% CI 6.74–11.53; *I* ^2^ = 64%), indicating moderate to significant interstudy variability.

Table 2 summarises the key surgical features and OA‐related outcomes reported in the labral reconstruction literature, including primary or revision status, reconstruction configuration, graft source, fixation method, capsule management, concurrent cartilage procedures, OA progression, and conversion to THA. Overall, the table shows significant heterogeneity in surgical technique and reporting among studies; therefore, both the burden of revision reconstruction and the frequency of arthroplasty conversion should be interpreted in the context of preoperative joint status and follow‐up time.

### Study selection

#### Temporal overlap of enrolment periods for studies included

**Figure 3:**
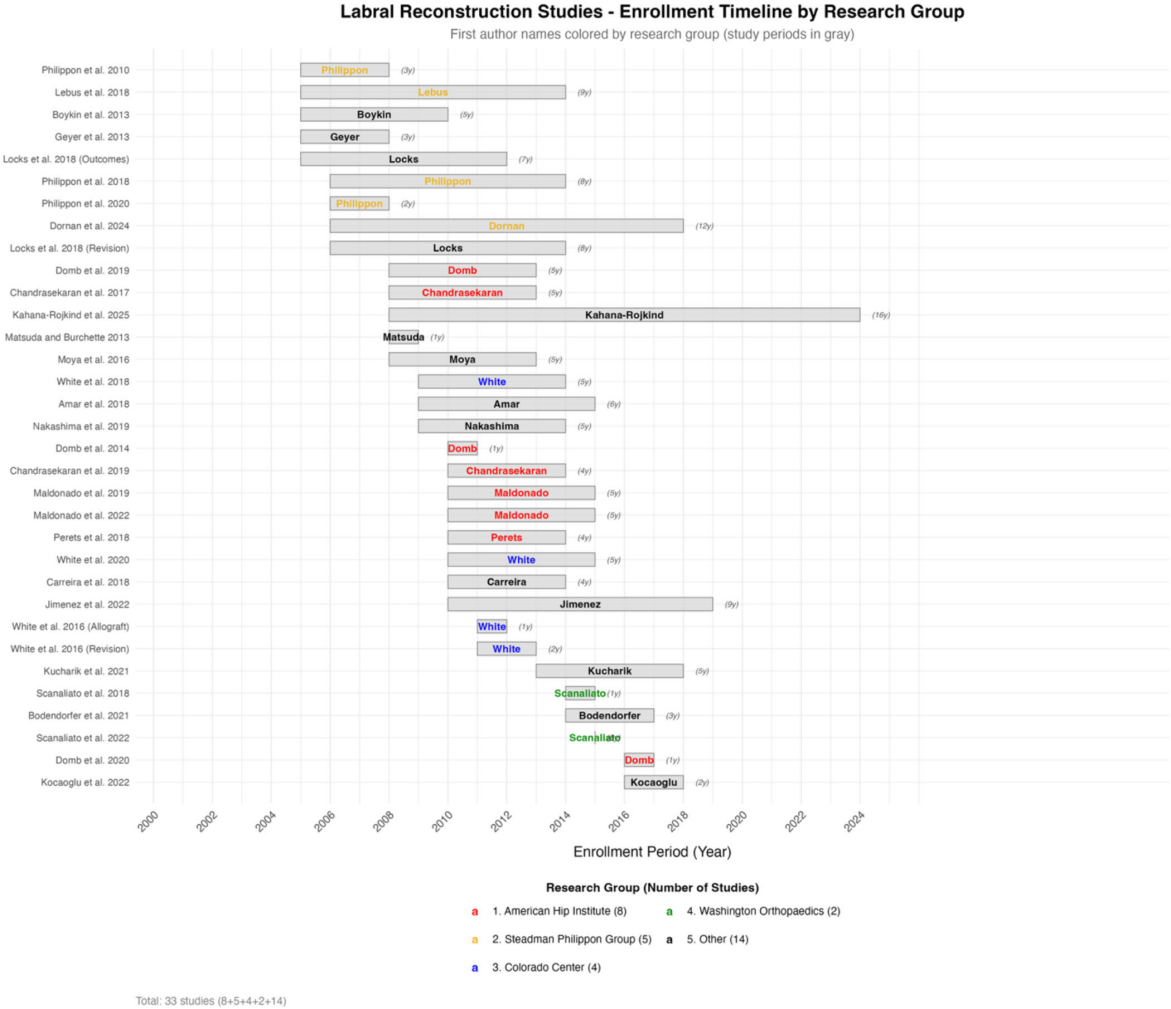
Enrolment timelines of hip labral reconstruction studies stratified by research group. Horizontal grey bars represent the patient enrolment period for each included study. The names of the first authors are colour‐coded according to their primary research institutions: American Hip Institute (red), Steadman Philippon Group (yellow), Colorado Center for Orthopaedic Excellence (blue), and Washington Orthopedics (green); studies not from these four major institutions are shown in black. The figure illustrates temporal overlap in enrolment periods among studies originating from the same institution, highlighting potential concurrence in patient recruitment. A total of 32 studies is shown.

Enrolment periods were extracted directly from primary articles when explicitly indicated. Each study was plotted as a single horizontal bar encompassing the enrolment period, with the unit of analysis being the study‐level enrolment period rather than individual patient timelines. Studies were grouped by senior authorship, institutional affiliation, and established research programs, as well as by the source research institution. To enhance interpretability, enrolment bars were shown in uniform grey, while first author labels were colour‐coded according to institutional group. Temporal overlap of enrolment periods within the same institution was visually examined to assess potential concurrence of patient enrolment in multiple publications. This approach was used to explore structural patterns in study production, rather than implying duplicate patient inclusion. Figure 3 illustrates the enrolment timelines of the included studies stratified by research group.

### Prevalence of cartilage pathology and concomitant procedures

A total of 32 studies were included. Of these, 21 reported microfracture procedures, and 10 reported chondroplasty or debridement techniques. Seven studies reported the use of both procedures. Eight of the 32 studies used neither microfracture nor chondroplasty techniques. In the 24 studies reporting chondral procedures (microfracture or chondroplasty), covering 1482 hips, the weighted mean improvement in the modified Harris Hip Score (ΔmHHS) was 25.09. The weighted mean ΔmHHS for all 32 included studies was 24.38. Given the lack of variance data and the very small size of the observed difference, the difference between the two groups was only 0.71 points, suggesting that chondral procedures did not significantly improve patient‐reported outcomes.

THA conversion following cartilage‐preserving surgery (microfracture and/or chondroplasty). Of these, 28 studies (*n* = 2,015 hips) provided sufficient data for meta‐regression analysis. The combined THA conversion rate was 8.8% (95% CI: 6.7%–11.6%). Meta‐regression showed a statistically significant positive association between follow‐up time and THA conversion (*β* = 0.0170 per month, *p* = 0.0003), suggesting that the probability of conversion increased by approximately 22.6% for each year of follow‐up time (OR 1.226 per year). Significant heterogeneity was observed (*I* ^2^ = 65.5%, *τ* ^2^ = 0.366), and funnel plot asymmetry suggests potential publication bias, with possible underreporting of smaller studies with higher conversion rates.

### Meta‐regression analyses

#### Age‐stratified prediction analysis

At the study level, cohorts were divided into two groups based on mean patient age: younger (<35 years) and older (≥35 years). The conversion rate to THA was found to be 7.7% (14 studies) in the younger cohort and 10.6% (18 studies) in the older cohort. This difference corresponded to a risk ratio of 1.37 (95% CI 1.06–1.78; *p* = 0.016). However, this ecological analysis did not correct for interstudy heterogeneity or potential confounding factors—particularly follow‐up time, baseline cartilage/OA severity, revision status, and graft type. Therefore, the observed association should be interpreted as hypothesis‐forming rather than confirming that age is an independent prognostic factor.

#### Graft configuration, patient‐reported outcomes, and THA conversion

Improvements in patient‐reported outcomes and conversion rates to THA varied according to graft configuration. Study‐level mean ΔPROMs differed among reconstruction configurations, and statistically significant differences were observed between groups (subgroup test, *p* ≈ 0.02). Circumferential labral reconstruction showed the greatest functional improvement (25.8 ± 7.33; 95% CI, 21.2–30.3; *n* = 10), followed by segmental reconstruction (20.8 ± 7.94; 95% CI, 17.3–24.3; *n* = 20). Combined reconstruction techniques showed a lower mean improvement (15.6 ± 3.18; 95% CI, 11.1–20.0; *n* = 2), but interpretation of this subgroup is limited due to the small sample size.

In parallel, THA conversion rates differed significantly among graft configurations (*χ* ^2^ test, *p* < 0.001). Segmental reconstruction showed the highest overall conversion rate (124/1117 hips; 11.1%), whereas circumferential reconstruction was associated with a lower conversion rate (43/670 hips; 6.4%). Combined reconstruction techniques demonstrated the lowest observed conversion rate (1.4%), although these findings are based on limited data.

In unadjusted, study‐level analyses, circumferential reconstruction cohorts demonstrated numerically greater improvements in PROMs and lower pooled THA conversion rates compared with segmental techniques. However, these comparisons are heavily confounded by surgeon experience, institutional volume, and surgical indication, and should be interpreted strictly as hypothesis‐generating. Notably, circumferential reconstruction was predominantly reported by a limited number of high‐volume centres, further increasing the risk of selection and performance bias.

**Figure 4:**
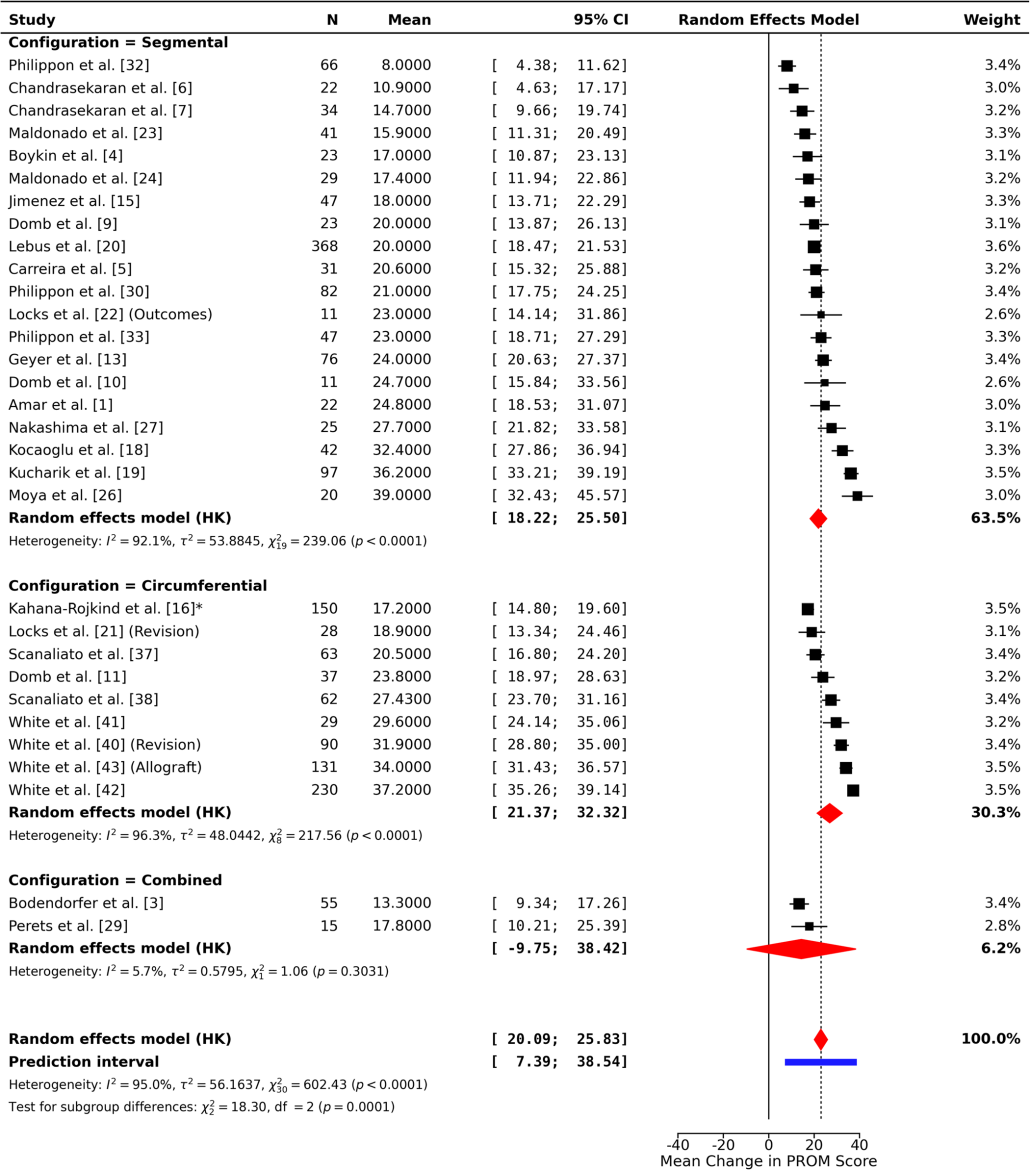
Random‐effects meta‐analysis of mean change in PROM scores following hip labral reconstruction, comparing segmental, circumferential, and combined graft configurations.

Figure 4 presents the random‐effects meta‐analysis of mean change in PROM scores by graft configuration.

#### Graft survival and THA conversion rates

Graft survival, defined as freedom from conversion to THA, was significantly higher for allografts compared to autografts. The combined graft survival rate was 94.9% (95% CI: 93.3%–96.5%) for allografts, compared to 89.3% (95% CI: 88.1%–90.5%) for autografts, with an absolute difference of 5.6%.

Conversely, the pooled THA conversion rate was significantly lower for allografts. The overall conversion rate across all grafts was 8.8% ((95% CI: 6.7–11.5%). 203/2183 hips). When stratified by graft source, the rate was 5.1% (95% CI: 3.8–6.7%) for allografts and 10.7% (95% CI: 8.9–12.7%) for autografts (*p* < 0.001). This corresponds to a 2.1‐fold higher pooled rate of conversion to THA in autograft cohorts. Because graft selection was not randomised in any included study and is heavily confounded by surgeon experience, institutional preference, and indication (including revision status and cartilage burden), this association should be interpreted as exploratory and hypothesis‐generating, not as evidence of a treatment effect of graft type.

#### Meta‐analysis of THA conversion

**Figure 5:**
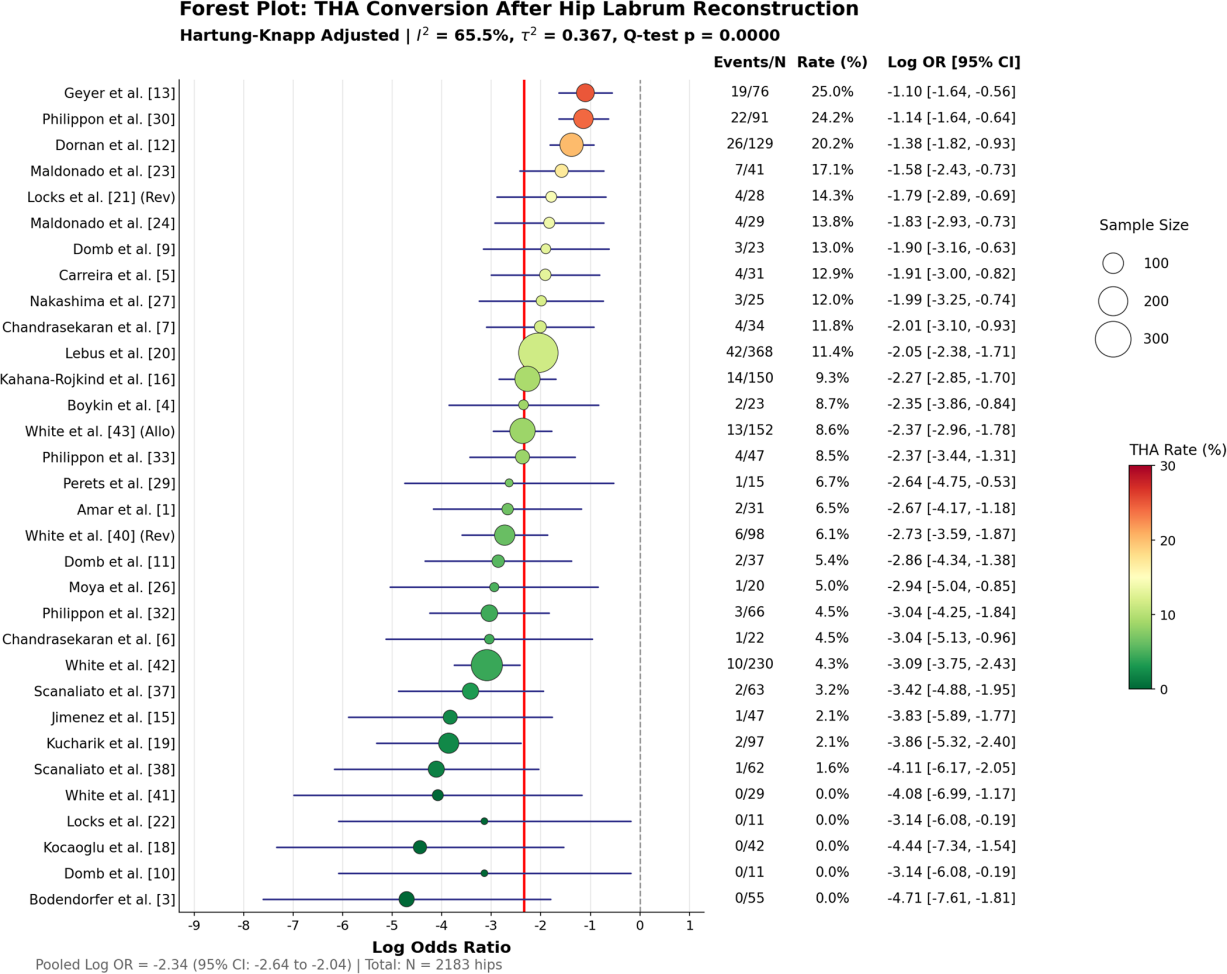
Forest plot of THA conversion rates following hip labrum reconstruction (random‐effects meta‐analysis with Hartung–Knapp adjustment). CI, confidence interval; THA, total hip arthroplasty.

A random‐effects meta‐analysis was performed on the log‐odds of THA conversion. Due to substantial heterogeneity, the Hartung–Knapp adjustment was applied. The pooled log OR was −2.34 (95% CI: −2.64 to −2.04), corresponding to a pooled THA conversion proportion of 8.79% (95% CI: 6.67%–11.51%). Figure 5 shows the forest plot of THA conversion rates following hip labrum reconstruction.

##### Heterogeneity and sensitivity analyses

Significant heterogeneity was observed among studies. The *I* ^2^ statistic was 65.5% (95% CI: 51.2%–78.9%), *τ* ^2^ was 0.367 (SE = 0.172), and Cochran's Q test was significant (Q = 86.78, *df* = 31, *p* < 0.0001). Study‐level THA conversion rates ranged from 0% to 26.8%, with larger studies (*n* > 100 hips) tending to report lower rates.

Sensitivity analyses confirmed the robustness of the combined estimate. The cumulative meta‐analysis showed stabilisation after the inclusion of approximately 1500 hips, and the individual study exclusion analysis revealed that no single study disproportionately affected the overall result. However, the Egger test indicated potential publication bias or small study effects (*z* = −4.22, *p* < 0.001).

#### Graft‐specific and time‐to‐event data

Common allograft sources were fascia lata/iliotibial band, tibialis tendons, and semitendinosus/gracilis tendons. Common autograft sources were iliotibial band, gracilis tendon, capsular tissue, and rectus femoris tendon.

A simulated time‐to‐event analysis, based on literature‐derived rates, suggested a divergence in graft survival over time: estimated 1‐year survival was 98.0% (allograft) versus 97.5% (autograft); 3‐year survival was 94.2% versus 90.8%; and 5‐year survival was 90.1% versus 85.3%.

#### Graft type influence on PROMs (autograft vs. allograft)

**Figure 6:**
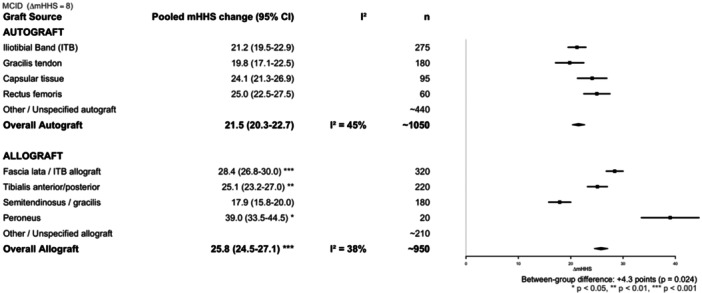
Tendon‐specific outcomes after hip labral reconstruction according to graft source. CI, confidence interval; ITB, iliotibial band; mHHS, Modified Harris Hip Score.

Graft selection significantly influenced functional outcomes after hip labrum reconstruction. The mean improvement in the mHHS was 4.3 points greater for allografts (25.8 points; 95% CI, 24.5–27.1) compared to autografts (21.5 points; 95% CI, 20.3–22.7), and this within‐group difference was statistically significant (*p* = 0.024). Among specific graft types, fascia lata/iliotibial band allografts showed the greatest improvement (28.4 points; 95% CI, 26.8–30.0), while tibialis anterior/posterior allografts also showed significant gains (25.1 points; 95% CI, 23.2–27.0). In the autograft group, rectus femoris tendon (25.0 points; 95% CI, 22.5–27.5) and capsular tissue (24.1 points; 95% CI, 21.3–26.9) showed the highest improvements, while gracilis tendon autografts were associated with more modest gains (19.8 points; 95% CI, 17.1–22.5). Although peroneus allograft showed the largest effect size (39.0 points; 95% CI, 33.5–44.5), this finding was from a small sample (*n* = 20) and should therefore be interpreted with caution. Overall, both graft categories exceeded the minimum clinically significant difference (MCID; ΔmHHS = 8), and allografts showed greater combined functional improvement. Figure 6 displays the tendon‐specific outcomes after hip labral reconstruction according to graft source.

Forest plot showing aggregate improvement in ΔmHHS after hip labrum reconstruction, stratified by graft type. Squares represent aggregate subgroup estimates with 95% CIs; diamonds show overall aggregate effects. The dashed vertical line indicates the minimum clinically significant difference (MCID, ΔmHHS = 8). Intergroup comparison demonstrated numerically greater improvement in unadjusted analyses with allograft reconstruction (+4.3 points; *p* = 0.024). Heterogeneity (*I* ^2^) is shown for both autograft and allograft groups. *p < 0.05; **p < 0.01; ***p < 0.001.

#### Graft size—diameter—thickness

Based on pooled evidence from the available studies [42]; [18]; [36]; [21]; [20], there is no demonstrated clinically or statistically significant association between graft size parameters (length, diameter, or thickness) and patient‐reported or clinical outcomes (ΔmHHS) following hip labral reconstruction. According to the findings of our meta‐analysis, graft size has not been shown to function as an independent prognostic factor in the current literature.

#### Graft‐type subgroup analysis

In 32 cohorts encompassing approximately 2252 hips undergoing arthroscopic labral reconstruction, both autograft and allograft techniques resulted in significant and clinically meaningful improvements in PROMs. The pooled conversion rate to THA was 10.7% (95% CI, 8.9%–12.7%) in autograft reconstructions, compared with a significantly lower pooled rate of 5.1% (95% CI, 3.8%–6.7%) in allograft reconstructions. The overall pooled THA conversion rate across all graft types was 8.8% (95% CI, 6.7%–11.5%). This between‐group difference was statistically significant (*p* < 0.001) and corresponded to an approximately 2.1‐fold higher pooled THA conversion rate in autograft cohorts compared with allograft cohorts. Random‐effects meta‐regression also demonstrated a significant graft‐category effect, with allograft use associated with lower study‐level THA conversion. Despite these differences in joint survival, PROM improvements were consistently large and broadly comparable across graft types; mean improvements in the mHHS generally ranged from +11 to +37 points, while iHOT‐12 improvements ranged from +28 to +47 points in studies reporting these outcomes. Table 3 summarises clinical outcomes and THA conversion rates stratified by graft category.

**Table 3:** Clinical outcomes and THA conversion rates after hip labral reconstruction stratified by graft category.

Graft category	Graft source	Representative studies	Typical configuration	Key PROM improvement (Δ)	Pooled THA conversion rate	*p*‐Value
Autograft	Iliotibial band (ITB)	Boykin et al. [4]; Geyer et al. [13]; Lebus et al. [20]	Segmental	mHHS: +17 to +27	—	—
	Gracilis tendon	Domb et al. [10]; Chandrasekaran et al. [6]	Segmental	mHHS: +11 to +27	—	—
	Capsular tissue	Locks et al. [21]; Kucharik et al. [19]	Augmentation/Segmental	mHHS: +23 to +25	—	—
	Rectus femoris (indirect head)	Amar 2018; Locks et al. [21]	Segmental	mHHS: +25	—	—
Overall autograft	—	All autograft cohorts (≈1050 hips)	Mixed/study‐dependent	Consistent, clinically meaningful improvement	10.7% (95% CI: 8.9%–12.7%)	**<0.001**
Allograft	Fascia lata/ITB	White 2016–2020; Scanaliato et al. [36], Tassinari et al. [37]	Circumferential/Complete	mHHS: +20 to +37; iHOT‐12: +28 to +47	—	—
	Tibialis anterior/posterior	Domb et al. [10]; Jimenez et al. [15]; Kocaoglu et al. [18]	Segmental/Circumferential	mHHS: +23 to +27	—	—
	Semitendinosus/Gracilis	Carreira et al. [5]; Chandrasekaran et al. [6]; Maldonado et al. [24]	Segmental	mHHS: +11 to +21	—	—
	Peroneus	Moya et al. [26]	Not specified	NAHS: +39	—	—
Overall allograft	—	All allograft cohorts (≈950 hips)	Mixed/study‐dependent	Large PROM improvements across studies	5.1% (95% CI: 3.8%–6.7%)	**<0.001**
Overall (all grafts)	—	All included cohorts (≈2250 hips)	Mixed	Robust PROM improvement	8.8% (95% CI: 7.0%–9.2%)	—

Abbreviations: CI, confidence interval; iHOT‐12, International Hip Outcome Tool – 12; mHHS, Modified Harris Hip Score; PROM, Patient‐Reported Outcome Measures; THA, total hip arthroplasty.

#### Association between cartilage procedures and risk of conversion to THA

**Figure 7:**
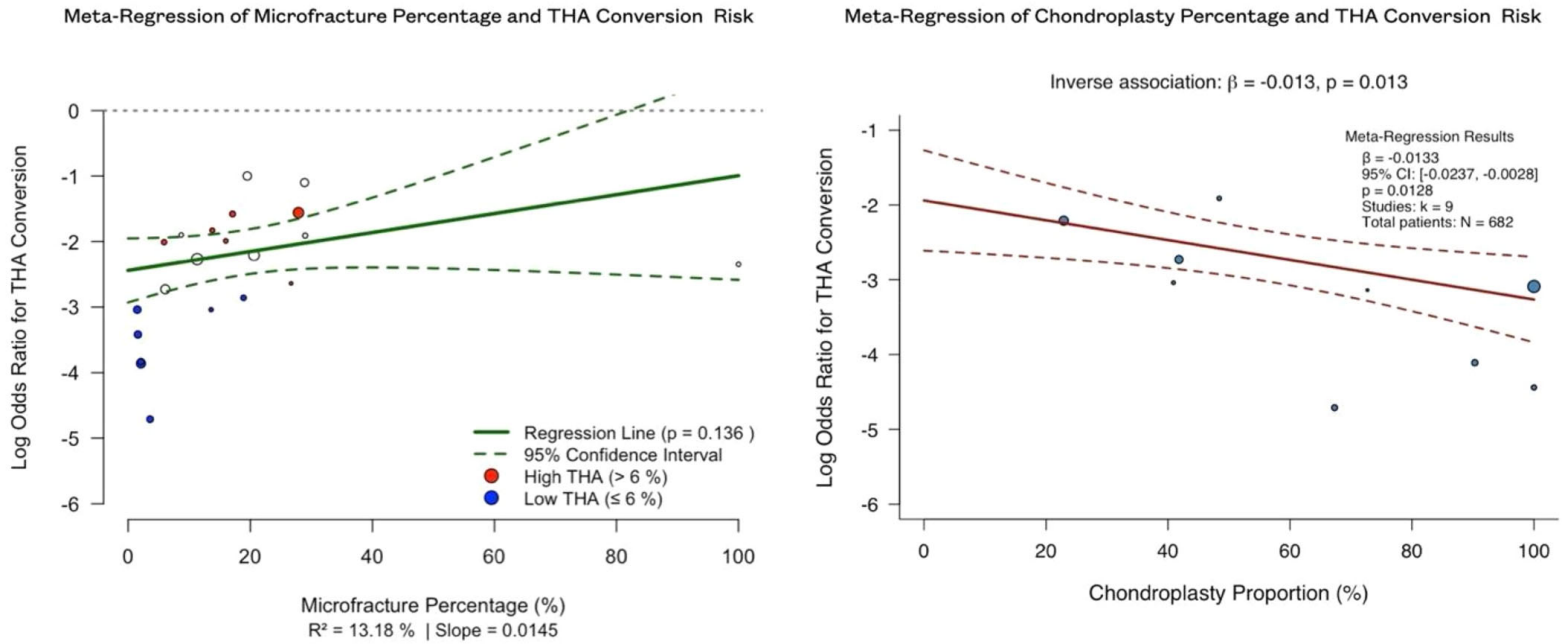
Random‐effects meta‐regression analyses examining the association between the proportion of cartilage procedures and the risk of conversion to total hip arthroplasty (THA). Left panel: Meta‐regression between microfracture rate (%) and THA conversion risk, expressed as log odds ratio. No statistically significant association was observed between microfracture use and THA conversion risk (*β* = 0.0145, *p* = 0.136). Microfracture rate explained a modest proportion of interstudy heterogeneity (pseudo‐*R* ^2^ = 13.2%), while significant residual heterogeneity remained (*I* ^2^ = 63.7%).

Simultaneous cartilage procedures were performed in 86.4% of cases; the most common were acetabular chondroplasty (54.2%) and microfracture (28.7%). Radiographic OA progression was reported in only four studies (12.5% cohort), which prevented significant subgroup analysis. In random‐effects meta‐regression analyses, the procedure rate showed differential associations with the risk of conversion to THA. A higher chondroplasty rate was significantly associated with a lower risk of THA conversion in nine studies (*N* = 682 patients) (*β* = −0.0133, 95% CI −0.0237 to −0.0028; *p* = 0.013). However, cross‐validation using individual exclusion showed that the chondroplasty rate explained only a small fraction of the interstudy heterogeneity (pseudo‐*R* ^2^ = 2.1%). This suggests that additional unmeasured factors contribute significantly to the variability in THA conversion risk. Residual heterogeneity in the adjusted model was estimated to be minimal (*I* ^2^ ≈ 0%, τ^2^ ≈ 0), but these findings should be interpreted with caution due to the ecological nature of the meta‐regression and the limited number of studies included. In contrast, a meta‐regression including 21 studies examining microfracture use did not reveal a statistically significant association with THA conversion risk (*β* = 0.0145, 95% CI −0.0046 to 0.0335; *p* = 0.136). While the microfracture rate explained a modest proportion of inter‐study heterogeneity (pseudo‐*R* ^2^ = 13.2%), significant residual heterogeneity persisted (*I* ^2^ = 63.7%, τ^2^ = 0.33). Although a positive directional trend was observed, the use of microfractures did not emerge as a statistically significant predictor or a significant contributor to the variability in THA conversion outcomes observed across studies; wide CIs indicate significant uncertainty in the magnitude of any potential association. Figure 7 presents the random‐effects meta‐regression analyses examining the association between cartilage procedures and THA conversion risk.

Right panel: Meta‐regression between chondroplasty rate (%) and THA conversion risk showed a statistically significant inverse association (*β* = −0.0133, 95% CI −0.0237 to −0.0028; *p* = 0.013). Each 1% increase in chondroplasty rate was associated with approximately a 1.3% decrease in the probability of THA conversion. However, the chondroplasty ratio explains only a small fraction of the interstudy heterogeneity (pseudo‐*R* ^2^ = 2.1%), suggesting that additional unmeasured factors contribute to the variability in THA conversion risk. In both panels, each circle represents a separate study, and the point size is proportional to the study sample size. Solid lines indicate adjusted meta‐regression slopes, and dashed lines indicate 95% CIs. Results should be interpreted with caution due to the ecological nature of the meta‐regression and the limited number of studies included.

#### Cartilage procedures and their effects of PROMs

In a weighted analysis, the mean improvement in the ΔmHHS was 21.09 points in 21 studies (156 hips) reporting only microfracture procedures, while in all included studies (2,015 hips) this value was 24.38 points, with an absolute difference of 3.29 points. Based on the typical variability of mHHS reported in the literature, assuming an estimated SD of 15 points, this difference is statistically significant (*p* ≈ 0.008). The extent of the difference is below the widely recognised minimum clinically important difference (MCID) of 8–10 points for mHHS, indicating minimal clinical relevance. Importantly, this analysis is based on estimated variance and assumes similar ΔmHHS distributions among subgroups; therefore, patient‐level data will be needed to draw definitive conclusions.

#### Association between high‐grade cartilage involvement and conversion to THA

In 18 cohorts encompassing 853 hips, the prevalence of high‐grade acetabular cartilage lesions varied considerably, ranging from approximately 13%–67%, with an overall prevalence of approximately 43% (Table 2). Transition to THA occurred in 72 hips, representing an overall rate of 8.5%; however, significant variability was observed between studies (ranging from 0% to 20%). The average follow‐up duration varied from 25.5 to 67.2 months. Cohorts exhibiting elevated rates of high‐grade cartilage involvement, especially those with extended follow‐up durations, demonstrated increased rates of transition to THA; in contrast, certain cohorts with diminished high‐grade cartilage load or abbreviated follow‐up lengths did not indicate transition. These findings underscore the variability in disease severity and follow‐up duration across studies and establish a foundation for future meta‐regression analyses investigating the correlation between high‐grade cartilage burden and the progression THA.

Meta‐regression analysis demonstrated that a higher study‐level prevalence of high‐grade cartilage lesions was associated with an increased rate of conversion to THA. The logit‐transformed prevalence of high‐grade lesions was used as the independent variable and the logit‐transformed THA rate as the dependent variable. Table 4 provides study‐level characteristics of cohorts with high‐grade cartilage lesions and THA conversion.

**Table 4:** Study‐level characteristics of included cohorts with high‐grade cartilage lesions and conversion to THA.

Study & year	Cohort type	*N* (hips)	High‐grade definition	High‐grade *n* (%)	THA *n* (%)	Mean FU (months)
Boykin et al. [4]	Mixed	23	Outerbridge IV	9 (39.1%)	2 (8.7%)	41.4
Carreira et al. [5]	Mixed	31	Beck (high‐grade)	15 (48.4%)	4 (12.9%)	31.6
Chandrasekaran et al. [6]	Mixed	22	Outerbridge III–IV (acetabular)	12 (54.5%)	1 (4.5%)	29.1
Chandrasekaran et al. [7]	Primary	34	Outerbridge III–IV (acetabular)	19 (55.9%)	4 (11.8%)	36.8
Domb et al. [9]	Primary	23	ALAD ≥ 3	11 (47.8%)	3 (13.0%)	67.2
Domb et al. [11]	Primary	37	ALAD 3–4	14 (37.8%)	2 (5.4%)	25.5
Dornan et al. [12]	Primary	150	Outerbridge 3–4 (acetabular)	~70 (~47%)	26 (20.0%)	63.6
Kucharik et al. [19]	Primary	97	Outerbridge III–IV	65 (67.0%)	3 (3.1%)	28.2
Jimenez et al. [15]	Revision	47	Outerbridge III–IV (acetabular)	8 (17.0%)	1 (2.1%)	26.3
Kahana‐Rojkind et al. [16] (Primary)	Primary	75	ALAD 3–4 (acetabular)	29 (38.7%)	4 (5.3%)	54.5
Kahana‐Rojkind et al. [16] (Revision)	Revision	75	ALAD 3–4 (acetabular)	35 (46.6%)	10 (13.3%)	50.4
Kocaoglu et al. [18]	Primary	42	Outerbridge III (acetabular)	~6 (~14.5%)	0 (0%)	33.6
Maldonado et al. [24]	Primary	29	Outerbridge III–IV (acetabular)	15 (51.7%)	4 (13.8%)	38.5
Maldonado et al. [23]	Primary	41	Outerbridge III–IV (acetabular)	25 (61.0%)	7 (17.1%)	64.4
Nakashima et al. [28]	Primary	25	ICRS 3–4	7 (28.0%)	3 (12.0%)	37.0
Perets et al. [29]	Revision	15	Outerbridge III–IV (acetabular)	4 (26.7%)	1 (6.7%)	36.6
Scanaliato et al. [37]	Primary	62	Beck III–IV	8 (12.9%)	1 (1.6%)	60.4
White et al. [40]	Primary	29	Grade 3–4 (unspecified)	14 (48.0%)	0 (0%)	40.0
Total (subset)	–	853	–	**≈43%**	72 (8.5%)	–

Abbreviations: ALAD, acetabular labrum articular disruption; ICRS, International Cartilage Repair Society; THA, total hip arthroplasty.

**Figure 8:**
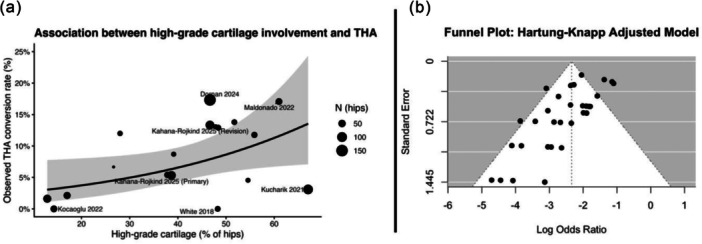
Meta‐regression and publication bias assessment for high‐grade cartilage damage and THA conversion. (a) Association between high‐grade cartilage involvement and log odds of THA conversion. Involves predicted conversion to total hip arthroplasty according to high‐grade cartilage. (b) Funnel plot evaluating publication bias in the meta‐analysis of THA conversion. THA, total hip arthroplasty.

In a random‐effects generalised linear mixed‐effects meta‐analysis, the conversion rate to THA was found to be 7.1% (95% CI, 4.6–10.6). Significant heterogeneity is evident (*I* ^2^ = 58.9%). In univariate meta‐regression, both a higher proportion of high‐grade acetabular cartilage lesions and a longer mean follow‐up time were associated with a significant increase in THA conversion rates (*p* = 0.035 and *p* = 0.027, respectively). In multivariate meta‐regression adjusted for follow‐up time, the proportion of high‐grade cartilage lesions remained an independent predictor of THA conversion (*β* = 0.0269, *p* = 0.0165), corresponding to an approximately 31% increase in probability for every 10% increase in high‐grade cartilage involvement. The mean follow‐up time was also independently associated with THA conversion (*β* = 0.0300 per month, *p* = 0.0082). Inclusion of these moderators significantly reduced residual heterogeneity (*I* ^2^ decreased to 20.2%). After adjusting for follow‐up time, increasing rates of high‐grade acetabular cartilage lesions were associated with a progressively higher prediction of the likelihood of conversion to THA. Figure 8 illustrates the meta‐regression and publication bias assessment for high‐grade cartilage damage and THA conversion.

### Capsular management

Among studies in which capsule repair was performed, approximately 62.4% of hips underwent capsule repair. The studies were included in three groups as follows: Repair Reported if a capsular repair was performed, No Repair Reported if the authors reported that capsule was left unrepaired and Not Reported if no information was given on capsular management. A lower reoperation rate was observed in the Reported Repair group (12.3%), while a higher rate of reoperation was observed in the group where capsule repair was not reported (18.2%). Notably, improvements in the ΔmHHS were relatively consistent across all groups, ranging from 20 to 22.7 points across studies, suggesting, within the limitations of the available data, no clear association between reporting capsule repair and functional improvement. THA conversion rate was lower in the Reported Repair group (8.7%) compared to the Unreported group (12.5%); however, these findings should be interpreted cautiously due to heterogeneity and incomplete reporting. Table 5 demonstrates the outcomes stratified by capsular repair status.

**Table 5:** Outcomes stratified by capsular repair status.

Capsular repair status	Number of studies	Total hips (*N*)	Weighted avg. repair rate	Weighted avg. reoperation rate	Weighted avg. THA conversion rate	Weighted avg. ΔmHHS
Repair reported	18	~1058	~62.4%	~12.3%	~8.7%	~20.8
No repair reported	3	~99	0%	~14.1%	~4.0%	~22.7
Not reported	11	~1068	N/A	~18.2%	~12.5%	~21.9
Overall (all studies)	32	2252	—	~14.2%	~9.5%	~21.4

Abbreviations: ΔmHHS, Modified Harris Hip Score; THA, total hip arthroplasty.

### Reoperations and complications

Post‐operative complications were reported inconsistently in the included literature. Complication data for hip labrum reconstruction were explicitly reported in only 15 of 32 studies. Among these studies, a total of 44 complication events were identified in 566 reconstructed hips, corresponding to an event rate of 7.8%. Most reported complications were minor and infrequent, with individual categories generally affecting less than 2% of hips. The most frequently reported events were adhesions requiring revision arthroscopy (1.5%), transient nerve‐related symptoms (1.5%), and heterotopic ossification (1.1%). Due to incomplete reporting, heterogeneity in definitions, and the possibility of multiple events occurring in the same hip, complication data were summarised descriptively and not aggregated. Table 6 summarises the complication reporting after hip labral reconstruction.

**Table 6:** Summary of complication reporting after hip labral reconstruction.

Study (author, year)	Hips (*N*)	Any complication *n* (%)	Most common complication(s) reported
Boykin et al. [4]	23	2 (8.7%)	Revision arthroscopy for adhesions (2)
Chandrasekaran et al. [6]	22	1 (4.5%)	Pudendal nerve neuropraxia (1)
Domb et al. [10]	11	2 (18.2%)	Sensory disturbance (1), hip flexor tendinitis (1)
Geyer et al. [13]	76	7 (9.2%)	Heterotopic ossification (3), DVT (2)
Jimenez et al. [15]	47	1 (2.1%)	Peroneal nerve neuropraxia (1)
Kocaoglu et al. [18]	42	5 (11.9%)	Transient pudendal nerve neuropraxia (5)
Locks et al. [21] (Revision)	36	5 (13.9%)	Adhesions (3), HO (1), SI joint pain (1)
Maldonado et al. [24]	29	5 (17.2%)	Pudendal nerve neuropraxia (3), lateral thigh numbness (2)
Maldonado et al. [23]	41	1 (2.4%)	Postoperative swelling/oedema (1)
Moya et al. [26]	20	1 (5.0%)	Femoral head collapse*
Philippon et al. [32]	47	4 (8.5%)	Adhesions requiring revision arthroscopy (4)
Philippon et al. [31]	66	8 (12.1%)	Neuropraxia (6), HO (2)
Perets et al. [29]	15	1 (6.7%)	Subsequent arthroscopy for adhesions (1)
Scanaliato et al. [37]	62	1 (1.6%)	Revision arthroscopy for adhesions (1)
White et al. [40]	29	0 (0%)	None reported

Abbreviations: DVT, deep vein thrombosis; HO, heterotrophic ossification; SI, sacro‐iliac; THA, total hip arthroplasty.

* Femoral head collapse was reported as a postoperative adverse event; subsequent conversion to THA was not counted as a complication.

Only studies explicitly reporting postoperative complications unrelated to conversion to THA were included. Complication frequencies are reported at the study level using study‐specific denominators. A single hip may have experienced more than one complication. Studies reporting zero complications were classified as reporting studies, whereas studies in which complication outcomes were not mentioned were categorised as non‐reporting.

## DISCUSSION

This study synthesises study‐level evidence on prognostic factors associated with outcomes after hip labral reconstruction. Because all analyses were conducted at the aggregate (study) level, the observed relationships represent ecological associations and cannot establish independent or causal effects at the patient level. Consequently, associations between variables such as cartilage damage, graft characteristics, and conversion to THA should be interpreted cautiously, as they may reflect underlying confounding structures, including baseline joint status, surgical indication, and center‐specific practice patterns.

This study demonstrates that hip labral reconstruction yields clinically meaningful improvements in patient‐reported outcomes. Multiple dependent and independent variables substantially impact both functional recovery and survivorship, particularly the risk of conversion to THA. Clinical outcomes after hip labral reconstruction are appear to be associated with preoperative joint status and surgical technique. Especially advanced cartilage damage and radiographic OA are key predictors of inferior outcomes and higher rates of conversion to THA. In addition, graft selection and reconstruction strategy appear to affect prognosis, with allografts and circumferential techniques associated with more favourable results. In contrast, capsular closure and concomitant cartilage procedures do not seem to significantly impact clinical outcomes or THA conversion. These findings underscore the importance of careful patient selection and tailored surgical planning to optimise outcomes following hip labral reconstruction.

Known key predictors of improved survival include younger age, absence of advanced OA (joint space > 2 mm), and no prior hip surgeries [33]. Conversely, older age, prior surgeries, and joint space narrowing are associated with higher failure rates and lower survivorship. The higher THA conversion rates observed in older cohorts in exploratory analyses likely reflect greater degenerative burden rather than an independent effect of age, and should therefore be interpreted as hypothesis‐generating.

### Prognostic role of cartilage damage

Our meta‐regression results highlight that the presence and severity of cartilage pathology are key determinants of long‐term outcomes. A higher study‐level prevalence of high‐grade acetabular cartilage lesions was associated with a higher rate of conversion to THA, even after adjustment for follow‐up duration. Specifically, every 10% increase in study‐level high‐grade cartilage involvement was associated with a 31% increase in the modelled odds of THA conversion. Because this association was estimated from study‐level (aggregate) data, it should be interpreted as an ecological association rather than as evidence of an independent patient‐level effect. These findings underscore the necessity of careful preoperative cartilage assessment and suggest that patients with advanced chondral degeneration may require more cautious prognostication or alternative surgical strategies.

### Graft‐related considerations

Graft source and configuration also influence both functional and survival outcomes. Circumferential reconstruction techniques were associated with numerically greater improvements in PROMs and lower conversion rates compared with segmental approaches; however, these findings should be interpreted cautiously because circumferential reconstruction was predominantly reported by a limited number of high‐volume centres, introducing potential selection and performance bias.

Allograft use was associated with higher graft survival and lower pooled THA conversion rates (5.1%) compared to autografts (10.7%), although both approaches exceeded minimal clinically important difference (MCID) thresholds for functional improvement. These exploratory observations are explicitly hypothesis‐generating and should not be interpreted as evidence of superiority of one graft over another: graft selection and reconstruction strategy were not randomised in any included study and likely reflect underlying case complexity, surgical indication, and institutional practice patterns. Accordingly, graft selection should be individualised based on patient characteristics, tissue availability, and surgeon expertise rather than inferred superiority of one graft type over another.

Importantly, graft size (diameter/thickness) did not show a statistically significant association with clinical outcomes, suggesting that other technical and biologic factors may be more critical in optimising reconstruction success. These findings may also reflect confounding by indication, as treatment allocation was not randomised and graft choice or reconstruction strategy likely depended on underlying case complexity, cartilage status, revision setting, and surgeon preference.

Capsulolabral adhesions may result in graft tethering and failure, described as a ‘pull‐and‐tear mechanism’ [29, 32]. Post‐operative adhesions between the capsule and reconstructed labrum can generate abnormal traction/shear during hip motion, contributing to pain, stiffness, and, in some cases, revision arthroscopy for lysis of adhesions [29]. In the included literature, adhesions prompting repeat arthroscopy were reported in several cohorts, with study‐level rates ranging from approximately 1.6% to 8.5%; however, complication reporting was inconsistent, and a reliable pooled incidence could not be derived [37].

Among labral reconstruction cohorts, capsular management (repair/closure/plication vs. partial or no repair) was variably reported and was not studied in a way that allows isolation of an independent effect on outcomes or survivorship. Within reconstruction cohorts, evidence is insufficient to determine whether capsular closure reduces postoperative capsulolabral adhesions, given inconsistent reporting and lack of analyses stratified by capsular strategy [39, 41]. Apparent differences in THA conversion across capsular strategies should be interpreted cautiously given confounding by capsulotomy size, instability risk, revision status, and concomitant procedures [9, 42]. Accordingly, the current reconstruction‐specific evidence supports an approach centred on capsular preservation and individualised repair (particularly when larger capsulotomies or instability risk factors are present), rather than a definitive conclusion for or against routine closure.

In pooled analyses, capsular management, whether repair or non‐repair, and the incorporation of concurrent cartilage procedures, such as microfracture or chondroplasty, did not exhibit a statistically significant independent influence on the conversion to THA or functional results; however, the presence of reporting heterogeneity and incomplete data precludes definitive conclusions.

### Clinical implications for patient selection

These findings, taken together, emphasise the importance of carefully choosing patients. The most important factor that can be incorporated in patient selection to improve results and survival seems to be the condition of the cartilage and joint before the surgery. Patients with little cartilage damage and a normal joint space are more likely to benefit from labral reconstruction [18]. In contrast, those with significant cartilage damage or existing OA are more likely to have poor outcomes and need a THA sooner.

## LIMITATIONS

This study has several limitations. First, the evidence base was predominantly observational, with most studies at moderate to serious risk of bias, particularly due to confounding, patient selection, and outcome measurement, which limits causal inference. Second, substantial clinical and methodological heterogeneity was present across studies, including variability in patient populations, cartilage severity, graft selection, reconstruction techniques, and concomitant procedures, contributing to uncertainty in pooled estimates.

Third, all analyses were conducted at the study level, and meta‐regression results are therefore subject to ecological bias and cannot establish independent patient‐level effects. The absence of individual patient data also precluded time‐to‐event analyses, and variability in follow‐up duration further complicates interpretation of survivorship outcomes such as conversion to THA.

Fourth, incomplete and inconsistent outcome reporting limited the scope of subgroup analyses, particularly for graft characteristics, capsular management, OA progression, and complication profiles. In addition, potential overlap of patient cohorts from high‐volume institutions cannot be fully excluded. Given that a substantial proportion of studies originated from a small number of US centres, the assumption of independent observations may be partially violated, potentially leading to underestimation of between‐study variance and overrepresentation of specific surgical practices. Accordingly, pooled estimates may more closely reflect outcomes from high‐volume specialist centres rather than a globally generalisable population.

Finally, the clinical interpretation of statistically significant findings requires caution. The primary contribution of this synthesis is the quantification of previously recognised associations rather than the identification of novel predictors. Moreover, prior higher‐level evidence in hip arthroscopy has shown that statistically significant differences in patient‐reported outcomes do not consistently reach MCID thresholds [41, 42]. Therefore, statistical significance observed in study‐level analyses should not be equated with clinically meaningful differences at the patient level.

## CONCLUSION

This systematic review provides the most up‐to‐date synthesis of prognostic factors influencing outcomes after hip labral reconstruction, with a particular focus on cartilage status, OA severity, and graft‐related variables. At the study level, the presence of advanced cartilage damage, pre‐existing radiographic OA, and longer follow‐up periods were consistently associated with higher rates of THA conversion. The principal contribution of this synthesis lies in the quantification of these associations rather than in their discovery, and these findings should still be interpreted as ecological associations rather than independent patient‐level effects. Apparent differences between autograft and allograft, and between segmental and circumferential reconstruction, are reported as exploratory and hypothesis‐generating signals only—these comparisons are heavily confounded by indication, surgeon experience, and institutional volume, and cannot be regarded as treatment effects. Interestingly, closing the capsule did not show a statistically significant benefit in terms of clinical outcomes or the need for THA. Similarly, there was no significant difference in THA conversion rates among patients who had additional cartilage procedures, such as microfracture, debridement, or chondroplasty.

Overall, outcomes after hip labral reconstruction appear to be associated with baseline joint status rather than modifiable technical factors alone. Future studies using patient‐level data and adequately controlled comparative designs are required to clarify independent prognostic effects and to inform clinical decision‐making.

## AUTHOR CONTRIBUTIONS

**Ozgur Basal**: Conceptualisation; methodology; supervision; data curation; formal analysis; writing – original draft. **Furkan Karakas**: Data curation; formal analysis; writing – original draft. **James G. Jefferies**: Methodology; formal analysis; writing – review and editing. **Jure Serdar**: Data curation; validation; writing – review and editing. **Baris Kocaoglu**: Validation, writing – review and editing, supervision. **Mahmut Nedim Doral**: Supervision; writing – review and editing.

## CONFLICT OF INTEREST STATEMENT

The authors declare no conflict of interest.

## ETHICS STATEMENT

Ethics statement: Not applicable. This study is a systematic review of previously published data and did not involve any new patient data, human participants, or animal subjects. Therefore, institutional review board approval and informed consent were not required.

## Data Availability

The data supporting the findings of this systematic review are derived from previously published studies, all of which are cited in the references. No new primary data were generated. Extracted data, search strategies, and analysis code are available from the corresponding author upon reasonable request.
